# Maximizing the Surface Sensitivity of LSPR Biosensors through Plasmon Coupling—Interparticle Gap Optimization for Dimers Using Computational Simulations

**DOI:** 10.3390/bios11120527

**Published:** 2021-12-20

**Authors:** Attila Bonyár

**Affiliations:** Department of Electronics Technology, Faculty of Electrical Engineering and Informatics, Budapest University of Technology and Economics, H-1111 Budapest, Hungary; bonyar@ett.bme.hu

**Keywords:** LSPR, nanoparticles, dimers, RIS, plasmon, nucleotide biosensors

## Abstract

The bulk and surface refractive index sensitivities of LSPR biosensors, consisting of coupled plasmonic nanosphere and nano-ellipsoid dimers, were investigated by simulations using the boundary element method (BEM). The enhancement factor, defined as the ratio of plasmon extinction peak shift of multi-particle and single-particle arrangements caused by changes in the refractive index of the environment, was used to quantify the effect of coupling on the increased sensitivity of the dimers. The bulk refractive index sensitivity (RIS) was obtained by changing the dielectric medium surrounding the nanoparticles, while the surface sensitivity was modeled by depositing dielectric layers on the nanoparticle in an increasing thickness. The results show that by optimizing the interparticle gaps for a given layer thickness, up to ~80% of the optical response range of the nanoparticles can be utilized by confining the plasmon field between the particles, which translates into an enhancement of ~3–4 times compared to uncoupled, single particles with the same shape and size. The results also show that in these cases, the surface sensitivity enhancement is significantly higher than the bulk RI sensitivity enhancement (e.g., 3.2 times vs. 1.8 times for nanospheres with a 70 nm diameter), and thus the sensors’ response for molecular interactions is higher than their RIS would indicate. These results underline the importance of plasmonic coupling in the optimization of nanoparticle arrangements for biosensor applications. The interparticle gap should be tailored with respect to the size of the used receptor/target molecules to maximize the molecular sensitivity, and the presented methodology can effectively aid the optimization of fabrication technologies.

## 1. Introduction

Localized surface plasmon resonance (LSPR)-based sensors use metallic nanoparticles to measure refractive index (RI) changes in their vicinity [1]. The plasmons—the collective oscillation of conductance electrons—are excited by incoming electromagnetic radiation, and their resonance frequency strongly depends on the dielectric properties of the medium surrounding the nanoparticles [2]. Changes either in the bulk refractive index of the surrounding liquid or gas or in molecular events such as bioreceptor–analyte binding on the surface of the nanoparticles cause a detectable redshift in their light extinction spectrum [3]. Several excellent review papers can be found that demonstrate how to utilize this effect in a large variety of sensing applications [4], from gas sensing [5] to chemical and biosensing [6,7], e.g., for biomarker [8,9,10] or nucleic acid detection [11,12].

To characterize and quantify the physical sensing performance of LSPR-based sensors, the two most commonly used parameters are the bulk refractive index sensitivity (RIS) and the figure of merit (FOM). RIS is defined as the change in the plasmon resonance wavelength (e.g., extinction spectrum peak shift) caused by the bulk refractive index change in the surrounding medium. FOM is the sensitivity divided by the bandwidth of the resonance peak (or full width at half maximum, FWHM) [13,14]. Since a higher physical RI sensitivity naturally means a higher molecular sensitivity as well, recent efforts in LSPR sensor development generally aim to increase and maximize RIS and FOM to reach an ultralow limit of detection (LOD) [15]. This is done by controlling the size, shape [16], composition (e.g., bimetallic [17] or core-shell structures [18]), and arrangement [19] of the particles. These factors elaborately interact with each other; thus, there are many strategies to improve the RIS of a plasmonic sensor (for a review on the topic, see [20]).

However, for biosensing applications, the molecular sensitivity of the sensor is primarily important, and the relationship between the physical RIS of an LSPR sensor and its molecular sensitivity or achievable molecular LOD is not trivial. It has been long known that, although the RIS of nanoparticle-based LSPR is usually around one order of magnitude smaller compared to the classic, thin-film-based SPR (the majority of LSPR sensors are in the 100–1000 nm/RIU range [16], compared to the ~3300 nm/RIU of SPR [21]) for biosensing applications, LSPR can match the molecular sensitivity of SPR [22]. This is attributed to the shorter plasmon field decay length of the nanoparticles, whose focused field makes them exceptionably well applicable for detecting molecular interactions on their surface [23].

Nevertheless, even for LSPR sensors, there still might be considerable differences in their RIS and molecular sensitivity. A recent review concerning nucleotide biosensors showed that LSPR sensors with nearly the same RIS reported vastly varying signals upon DNA hybridization [12]. For four sensors having a RIS around 100 nm/RIU, the reported maximal signal response (the total plasmon peak shift measured after DNA immobilization and subsequent hybridization with saturation concentration) was between 5 and 16 nm [23,24,25,26]. For sensors with sensitivities around 200 nm/RIU, the spread of the maximal response was between 9 and 33 nm [27,28].

The intensity and decay length of the plasmon field around the nanoparticles elaborately depends on the physical and geometrical properties [29,30]. Besides the size and shape of the nanoparticles, a significant contributor is the field enhancement and localization due to interparticle plasmonic coupling [31,32]. It was previously shown that decreasing the interparticle gap (and thus increasing the coupling between nanoparticles) leads to an increased bulk refractive index sensitivity [33,34,35,36]. More importantly, it was demonstrated that coupling focuses the field in the interparticle gap, thus effectively decreasing the plasmon decay length [37]. The decay length of nanoparticle arrangements can be characterized by the subsequent deposition of layers with known thickness and refractive index on the surface of the particles (both experimentally and by simulations) [38,39]. The effective response of the sensors to such layer depositions is often referred to as surface sensitivity [37], which can be considered more meaningful to describe the performance of LSPR sensors than bulk RIS, considering molecular sensing applications.

The aims of the current work are: first, to investigate the effect of plasmonic coupling on the bulk RI and surface sensitivity of coupled plasmonic dimers with simulation methods; secondly, to provide an answer to the controversy mentioned above between the reported RIS and the molecular LOD of nanoparticle-based sensors, and thirdly, to offer a convenient way to characterize the performance of coupled plasmonic nanoparticle systems by using the enhancement factor formulation (introduced in [33]). Numerical simulations were designed to model plasmonic nucleotide biosensors, where a thin layer of DNA/RNA molecules was bound to the surface of the nanoparticles. It is demonstrated that for affinity-type biosensing applications that work with thin layers of receptor and target molecules, tailoring the interparticle gap and thus optimizing coupling between the nanoparticles is essential to maximize the performance of the LSPR sensors.

## 2. Simulation Methods

**Figure 1:**
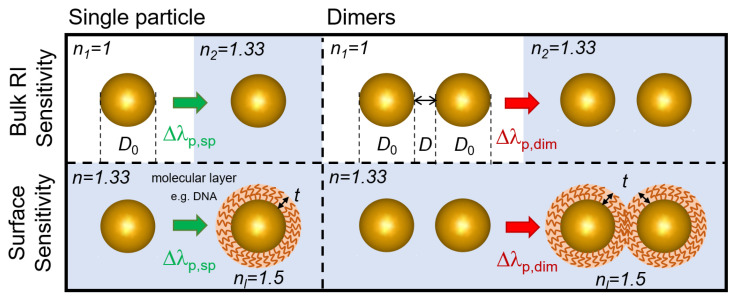
Illustration of the different simulation conditions and the definitions of bulk RI sensitivity (RIS) and surface sensitivity. Symbols: *D*_0_—nanosphere diameter, *D*—interparticle distance (gap), $\Delta \lambda_{p}$ —extinction peak shift (for single particles, sp, and dimers, dim), *t*—dielectric layer thickness, *n*_l_—dielectric layer refractive index.

All simulations were performed by using the boundary element method (BEM), as implemented in the form of the MNPBEM (Metallic NanoParticle Boundary Element Method) MATLAB Toolbox. For a detailed description of the toolbox, its advantages, and its accuracy compared to other numerical approaches, please see [40,41,42]. The ‘retarded’ solver that solves the complete solution of Maxwell’s equations was selected to obtain the extinction cross-section spectra of the investigated nanoparticle arrangements. For this, a plane-wave excitation was used, with a light propagation vector perpendicular and a linear polarization vector parallel to the symmetry axis of the two-particle systems. This way, the coupled plasmon modes of the dimers were excited properly. The dielectric functions used to model the gold nanoparticles were based on the experimental results of McPeak et al. [43]. Spherical nanoparticles composed of 256 vertices were created with the embedded ‘trisphere’ function of the toolbox, and nano-ellipsoids were created by rescaling the axes by using the ‘scale’ function. The ellipsoids were defined by two axes and their ratio *c*, where *c* = diameter/thickness. For the two investigated cases (*c* = 1.5 and 2), their diameter (*D*_0_) was always the longer axis, and the coupling and interparticle gap (*D*) were defined in the longitudinal direction. The dielectric environments were modeled with a constant refractive index (e.g., air *n* = 1 and water *n* = 1.33). The molecular layers deposited on the nanoparticles were modeled as a dielectric shell of *n* = 1.5, corresponding well with the equivalent refractive index used to model dense DNA monolayers in an aqueous environment [25,27,44]. The different simulation conditions are illustrated in Figure 1.

The bulk refractive index sensitivity (defined in Equation (1) [1]) was calculated as the peak shift ( $\Delta \lambda_{p}$ ) of the extinction spectrum upon changing the RI of the dielectric medium from *n*_1_ = 1 to *n*_2_ = 1.33, both for single particles ( $\Delta \lambda_{p,sp}$ ) and for dimers ( $\Delta \lambda_{p,dim}$ ). Contrary to RIS, where the RI change affects the whole accessible volume of the plasmonic electric field, surface sensitivity measures only a fraction of the field closest to the surface of the nanoparticles [37]. The surface sensitivity was obtained as the peak shift experienced upon adding a dielectric shell with a thickness of *t* and an RI of *n*_l_ = 1.5 in a constant dielectric environment of *n* = 1.33.

(1) $$RIS=\frac{\Delta \lambda_{p}}{\Delta n}$$

For the evaluation of the different nanoparticle dimers, the enhancement factor (*EF*) was used (introduced in [33]). The enhancement factor characterizes the improved performance of a multi-particle arrangement that originates from the plasmonic coupling between the particles, compared to uncoupled single particles with the same shape and size. It can be generally defined as the ratio of extinction peak shifts of the multi-particle and single-particle arrangements, as in Equation (2) [33], caused by the same modified RI conditions (either bulk RI change or layer deposition).

(2) $$EF=\frac{\Delta \lambda_{p,dim}}{\Delta \lambda_{p,sp}}$$

Since the enhancement factor can be used to characterize both bulk RI sensitivity and surface sensitivity, for precise annotation, we refer to the former as *EF*_∞_, while the *EF*_t=x_ form refers to an *EF* obtained by depositing a layer with a thickness of *t* = x nm on the nanoparticles. This annotation also indicates that the bulk *EF* is technically the same as the surface *EF* for a layer with a thickness of *t* = ∞.

## 3. Results and Discussion

**Figure 2:**
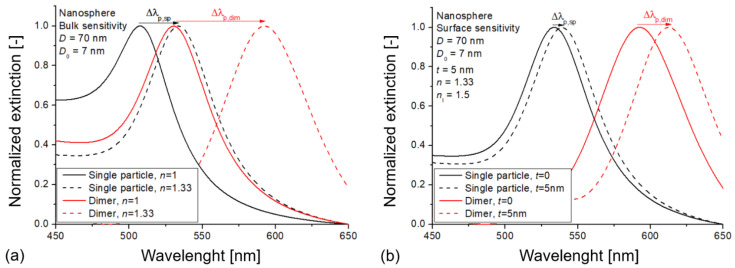
Normalized extinction spectra simulated on single-particle and dimer arrangements of gold nanospheres to illustrate the changes in bulk RI sensitivity (**a**) and surface sensitivity (**b**) caused by plasmonic coupling between the nanospheres. For the illustration of the different conditions, please see Figure 1.

To illustrate the effect of plasmonic coupling on the bulk RI and surface sensitivity of the nanoparticles, Figure 2 presents the normalized extinction spectra obtained for the various simulation conditions illustrated in Figure 1. Here, single gold nanospheres with a diameter (*D*_0_) of 70 nm were used, with a 7 nm interparticle distance (*D*) in the case of the dimers. This interparticle distance (*D*/*D*_0_ = 0.1) was small enough to properly excite the Σ coupled mode (in-phase bonding mode [45]) in the dimers that caused an increased peak shift upon the same RI change compared to single particles. The obtained extinction peak shifts for the bulk RI change were $\Delta \lambda_{p,sp}$ = 26.3 nm for a single nanosphere (*RIS*_sp_ = 78.9 nm/RIU) and $\Delta \lambda_{p,dim}$ = 62.6 nm for the dimers (*RIS*_dim_ = 187.8 nm/RIU), which yielded a bulk enhancement factor (*EF*_∞_) of 2.38. By depositing a dielectric layer of 5 nm thickness and *n*_l_ = 1.5, the experienced extinction peak shifts were $\Delta \lambda_{p,sp}$ = 6.1 nm and $\Delta \lambda_{p,dim}$ = 18.2 nm (Figure 2b), corresponding to an enhancement of 2.98 (*EF*_t=5nm_).

**Figure 3:**
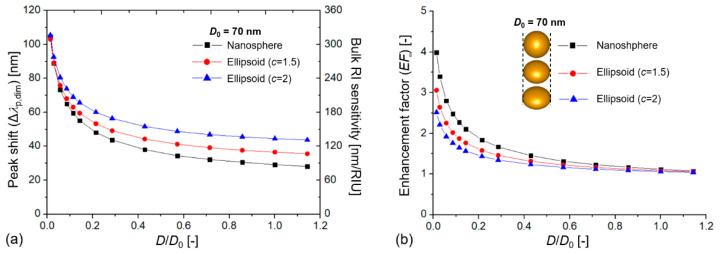
(**a**) Extinction peak shift of coupled plasmonic dimers ( $\Delta \lambda_{p,dim}$ ) and their calculated bulk refractive index sensitivity (RIS) as a function of the dimensionless *D*/*D*_0_ value, where *D* is the interparticle distance, and *D*_0_ is the particle diameter (*D*_0_ = 70 nm). (**b**) Calculated bulk enhancement factor (*EF*_∞_) values compared to single, uncoupled particles with the same size as a function of *D*/*D*_0_.

An *EF* > 1 indicates that the extinction shift signal origination from the RI changes around the particles was higher for the dimers than for the single particles owing to plasmon coupling. The enhancement increased with decreasing interparticle gap, as demonstrated in Figure 3 for nanospheres and ellipsoids (for raw data, see Table S1). The extinction peak shift, the bulk RI sensitivity, and the enhancement factor increased exponentially with a decreasing *D*/*D*_0_, which corresponds well with previous results on Au, Ag, In, and Si nanospheres and nanocubes [33,35,46].

An interesting aspect of the investigated nanodimers can be seen in Figure 3, namely, that although nano-ellipsoids had higher absolute peak shifts and RIS (with increasing *c*), their corresponding enhancement gain as a function of decreasing *D*/*D*_0_ was smaller. In this sense, the initial sensitivity advantage of nano-ellipsoids practically diminishes for *D*/*D*_0_ < 0.1.

**Figure 4:**
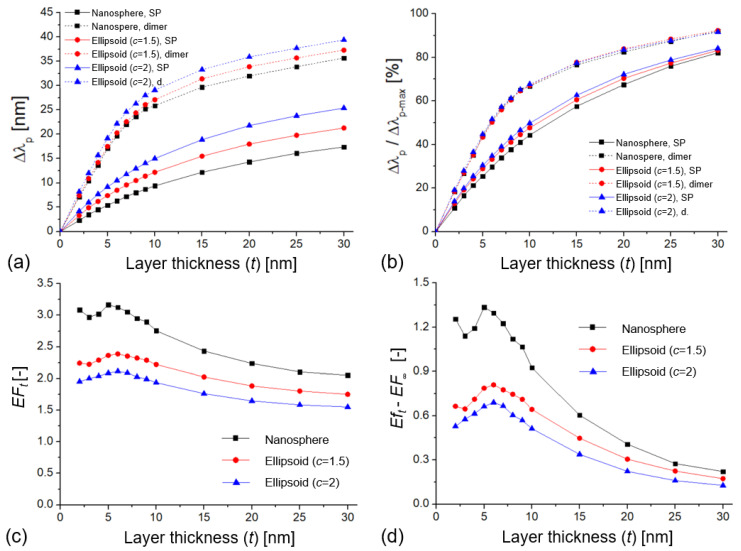
(**a**) Extinction peak shift ( $\Delta \lambda_{p}$ ) of different single-particle and dimer arrangements as a function of the deposited dielectric layer thickness (*n*_l_ = 1.5 in water, medium with *n* = 1.33). The diameter of the particles was 70 nm, with a 10 nm interparticle gap (*D*/*D*_0_ = 0.14). (**b**) The relative extinction peak shift as a function of the layer thickness where $\Delta \lambda_{p-max}$ was calculated as the peak shift upon changing the RI of the medium from 1.33 to 1.5. (**c**) Enhancement factor (*EF*_*t*_) as a function of the layer thickness. (**d**) Difference between surface and bulk enhancement factors (*EF*_*t*_ − *EF*_∞_) as a function of the layer thickness.

The open questions that originates from Figure 2 are how the enhancement factor can be different for bulk and surface sensitivities and how this could be used to maximize this effect to obtain higher signals upon using these nanoparticles as biosensors. To investigate this effect, the shift of the extinction peak was calculated as a function of an increasing layer thickness (*n*_l_ = 1.5 in water medium, with *n*_m_ = 1.33). The diameter of the particles was 70 nm, with a 10 nm interparticle gap (*D*/*D*_0_ = 0.14). As shown in Figure 4a, dimers of the same particle type always had a larger peak shift, consistent with their increased RIS due to plasmonic coupling. In Figure 4b, the relative extinction peak shifts were calculated as the ratio of $\Delta \lambda_{p}/\Delta \lambda_{p-max}$ , where $\Delta \lambda_{p-max}$ is defined as the peak shift upon changing the RI of the whole medium from 1.33 to 1.5 (or, in other words, coating the nanoparticles with a layer of infinite thickness, *t* = ∞).

Note that Figure 4a,b also characterize the decay length (*l*_d_) of the nanoparticles, which can be extracted from Figure 4a by using Equation (3) [37]. In our case, the three curves belonging to the single particles can be perfectly fitted with Equation (3) (R^2^ = 0.999), resulting in decay lengths of 33 nm (*c* = 1, nanosphere), 27.2 nm (*c* = 1.5, ellipsoid), and 25.4 nm (*c* = 2, ellipsoid). For single particles, the calculated *l*_d_ showed a negative correlation with both $\Delta \lambda_{p}$ and *c*, demonstrating that for nano-ellipsoids, the longitudinal mode focused the field, which accounted for higher peak shift and overall sensitivity.

(3) Δλ=RIS×(nl−nm)×(1−e−2tld)

Fitting the three curves in Figure 4a that belong to the coupled dimers, Equation (3) returned 16 nm (*c* = 1), 15.8 nm (*c* = 1.5), and 15 nm (*c* = 2). However, as can be seen in Figure 4a,b, these curves diverged from the ideal exponential relationship defined by Equation (3), which was originally introduced for thin-film-based propagating plasmons. The curves belonging to the dimer arrangements are clearly steeper, especially in the *t* ≤ *D*/2 region. Here a power law-like relation would better describe the curves (as was suggested, for example, in [47]), resulting in an even shorter decay length (between 11 and 12 nm).

The enhancement factors calculated based on Equation (2) are plotted in Figure 4c,d. All curves have a clear maximum at *t* = *D*/2 thickness, which was 5 nm in this case. The maximal *EF*_t_ values were 3.2, 2.4, and 2.1 for the three nanoparticle types, respectively. The practical meaning of *EF*_t_ is, for example, that dimer nanospheres of 70 nm diameter and 10 nm gap provide a 3.2 times higher signal compared to uncoupled spheres of the same size with *t* = 5 nm layer thickness. *EF*_∞_, on the other hand, is the ratio between the RIS of dimers (228.2 nm/RIU) and single particles (124.7 nm/RIU), which was ~1.8 for these nanospheres. As shown in Figure 4d, for small layer thicknesses, *EF*_t_ was higher than *EF*_∞_, which means an increased sensing performance for thin layers. Figuratively, the 3.2 vs. 1.8 difference in *EF*_t_ and *EF*_∞_ means that the coupled nanospheres with 228.2 nm/RIU RIS will respond to a 5 nm-thick layer as a single particle with 405.7 nm/RIU RIS would.

**Figure 5:**
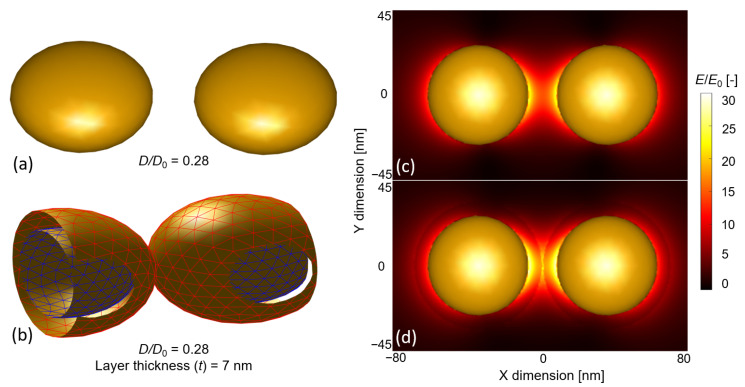
(**a**) An assembled nano-ellipsoid dimer in the MNPBEM toolbox, without layers in dielectric medium (*n* = 1.33). (**b**) Sectional perspective of the same nano-ellipsoid dimer with a deposited dielectric layer (*n*_l_ = 1.5) of 7 nm. The blue mesh represents the vertices of the gold ellipsoids, while the red mesh represents the surface of the dielectric layer. (**c**) The distribution of the electric field around the gold nano-ellipsoid dimer is illustrated in (**a**). The map represents the Z = 0 plane (top view). (**d**) Electric field distribution for the dimer with dielectric layers illustrated in (**b**).

The main message of Figure 4a,b is that the thin layers of dielectric materials can utilize the confined near field of the coupled particles more effectively. Depositing a 7 nm-thick layer on a single particle of 70 nm occupied only 34% of its plasmon field (Figure 4b). The same value was 56.7% for the coupled dimers, which corresponded to a 22.7% increase. The electric field between two coupled ellipsoids is plotted in Figure 5 without a dielectric layer (Figure 5c) and with a 7 nm-thick layer (Figure 5d), for the *D*/*D*_0_ = 0.28 case. It can clearly be seen that the 7 nm layer occupies the most sensitive regions (with the highest field strength) that are confined between the nanoparticles. By depositing thin layers on top of the nanoparticles, we worked in the most sensitive near-field region. Again, it has to be pointed out that Figure 4c,d showed a local maximum around *t* = *D*/2 thickness when the deposited layers touched and filled the interparticle gap. Above *t* = *D* (10 nm), the *EF* showed an exponential decay, and *EF*_t_ → *EF*_∞_ at *t* = ∞.

**Figure 6:**
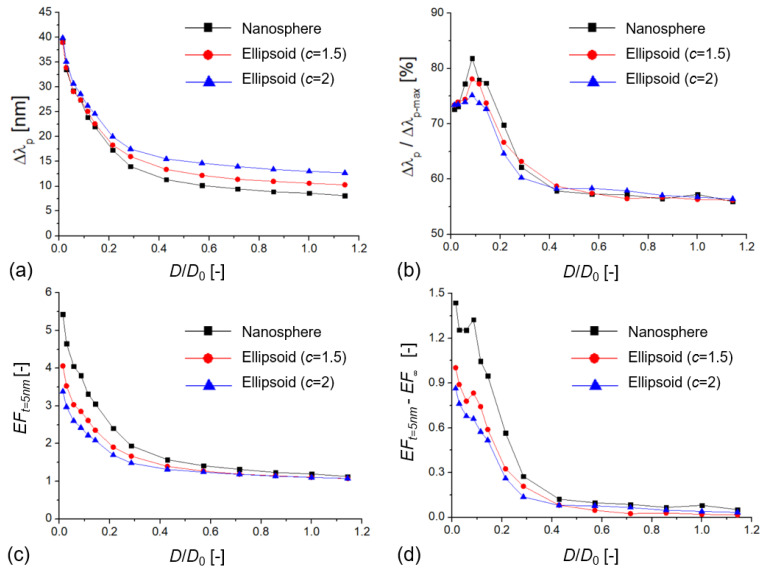
(**a**) Extinction peak shift ( $\Delta \lambda_{p}$ ) of different dimer arrangements as a function of the dimensionless *D*/*D*_0_ value (*D*_0_ = 70 nm) with a dielectric layer of 5 nm (*n*_l_ = 1.5 in water medium, with *n* = 1.33). (**b**) Relative extinction peak shift as a function of the dimensionless *D*/*D*_0_ value, where $\Delta \lambda_{p-max}$ is calculated as the peak shift upon the RI of the medium changing from 1.33 to 1.5. (**c**) Enhancement factor (*EF*_t=5nm_) as a function of *D*/*D*_0_. (**d**) Difference between surface and bulk enhancement factors (*EF*_t=5nm_ − *EF*_∞_) as a function of *D*/*D*_0_.

To investigate the parameter space further, Figure 6 presents simulation results where the deposited layer thickness was fixed at *t* = 5 nm, the nanoparticle diameter was *D*_0_ = 70 nm, and the interparticle gap was decreased, thus scaling the dimensionless *D*/*D*_0_. The enhancement factors were again calculated as the ratio of the presented dimers and single particles of the same size. The characteristic of the curves (exponential decay) is similar to what can be seen in Figure 3 for the bulk sensitivity tests, but a clear elevation of the left side of the curves is visible in Figure 6a,c. Based on Figure 6d, this is clearly the yield of the increased surface sensitivity. Again, please note that the curves in Figure 6b,d have a clear maximum, around *D*/*D*_0_ = 0.1–0.15, which translates to a 7–10 nm interparticle distance, which is also in the *t* = *D*/2 range. For nanospheres, in this region, the effective utilization of the plasmon field could reach around 80% (Figure 6b).

A few words should be spoken about the relevance of the investigated interparticle gaps and layer thicknesses, considering biosensing applications. For nucleotide sensors (utilizing DNA and RNA molecules) a 5 nm layer thickness would equal a fully extended ds-DNA of 15 base pairs, which is an entirely realistic receptor–target length for plasmonic applications (for more information, see a survey of plasmonic nucleotide sensors in [12]). For the optimization of nucleotide sensors, one should consider that ss-DNA chains that compose the empty receptor layer usually do not extend to their full length; thus, the effective layer thickness increases upon hybridization (ds-DNA chains are less flexible [48]). The optimization of immunosensors should consider the sizes of the antibody (in the 10 nm range) and the analyte, which can vary significantly between applications. Generally, it has to be noted that interparticle gaps below the *D* = *2 ∗ t* range might endanger the function of the sensor, since spatial confinement might prevent the proper binding of the analyte.

These results and recommendations are most relevant for fabrication technologies that have the possibility to fine-tune the interparticle gap of multi-particle arrangements. Nanoparticle gap engineering with sub-nanometer resolution was experimentally demonstrated in liquid phase for various shapes by using DNA linkers [49,50]. Particle arrays with controlled interparticle gaps can also be realized on substrates with lithography methods, such as electron beam, ion beam, or nanoimprint lithography [51], with fine control over the interparticle gap/diameter ratio (*D*/*D*_0_) in the 0.08–1 range [52]. A similar excellent control over the nanoparticle arrangement on large surface areas (cm^2^ range) was recently reported by using a template-assisted solid-state dewetting synthesis and subsequent nanoparticle transfer to a transparent polymer support [23].

It has to be mentioned that attaching the nanoparticles to solid supports will decrease their overall sensitivity, since a significant part of their near field is filled with the substrate material (with a constant refractive index). In the simulations for the investigated nanospheres and ellipsoids, this drop in sensitivity was between 10 and 15% (depending on the aspect ratio (*c*)), when a glass substrate (*n* = 1.51) was placed directly under the nanoparticles. For flat particles, such as nanodiscs, this effect can be higher. Although the substrate’s effect depends on the particle type and substrate material and should be investigated specifically for individual applications, due to the nature of the enhancement factor (see Equation (2)), for simple cases, it is not expected to modify the presented *EF* vs. *D*/*D*_0_ trends significantly.

## 4. Conclusions

A controversial aspect of LSPR biosensor development is that, although development efforts generally point toward maximizing the RIS of the sensors, sometimes nanosensors with lower RIS provide a higher sensor response when working with molecular layers below 10 nm, e.g., in nucleic acid sensors. It was demonstrated that by utilizing plasmonic coupling between nanoparticle dimers, the near field could be effectively confined, and the plasmon decay length shortened, which led to a significant surface sensitivity enhancement if the deposited layer thickness was comparable (favorably below) to the interparticle gap. As the simulation results on the nanospheres and nano-ellipsoids indicated, the response upon binding a molecular layer on the surface of the nanoparticle could be enhanced up to 3–4 times (compared to a single particle with the same size) by optimizing the interparticle gap and confining the plasmon field into the range of the layers. Generally, the response of the particles increased with a decreasing interparticle gap as their coupling intensified. A local maximum in sensor response (for a given layer thickness) was reached when the interparticle gap was twice the thickness of the layer. Here, ~80% of the sensor’s total dynamic range could be utilized.

As demonstrated, the enhancement factor formalism offers a convenient way to characterize and quantify the signal increase originating from plasmonic coupling of multi-particle nanostructures. The results and presented methodology can help optimize fabrication technologies to maximize the surface sensitivity of sensors, tailoring them to specific biosensing applications.

## Data Availability

The data presented in this study are available on request from the corresponding author.

## Supplementary Materials

The following are available online at https://www.mdpi.com/2079-6374/11/12/527/s1, Table S1: The raw data (simulation results) presented in Figure 3, Figure 4, and Figure 6.
