# Correction: Bonyár, A. Maximizing the Surface Sensitivity of LSPR Biosensors through Plasmon Coupling—Interparticle Gap Optimization for Dimers Using Computational Simulations. *Biosensors* 2021, *11*, 527

**DOI:** 10.3390/bios12060411

**Published:** 2022-06-14

**Authors:** Attila Bonyár

**Affiliations:** Department of Electronics Technology, Faculty of Electrical Engineering and Informatics, Budapest University of Technology and Economics, H-1111 Budapest, Hungary; bonyar@ett.bme.hu

## 1. Error in Figure/Table

In the original publication [1], there was a mistake in ***Figures 3 and 6*** as published. The unit of *D*/*D*_0_ (in the axes labels) is indicated as (nm), while it should correctly be dimensionless (-). The mistake only involves the figures; in the text and in the figure captions *D*/*D*_0_, was always correctly referred to as dimensionless. The corrected Figure 3 and Figure 6 appear below.

**Figure 3:**
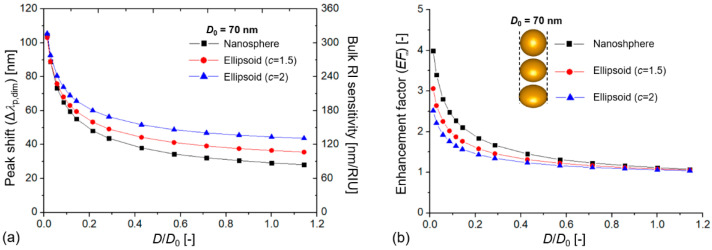
(**a**) Extinction peak shift of coupled plasmonic dimers ($\Delta \lambda_{p,dim}$) and their calculated bulk refractive index sensitivity (RIS) as a function of the dimensionless *D*/*D*_0_ value, where *D* is the interparticle distance, and *D*_0_ is the particle diameter (*D*_0_ = 70 nm). (**b**) Calculated bulk enhancement factor (*EF*_∞_) values compared to single, uncoupled particles with the same size as a function of *D*/*D*_0_.

**Figure 6:**
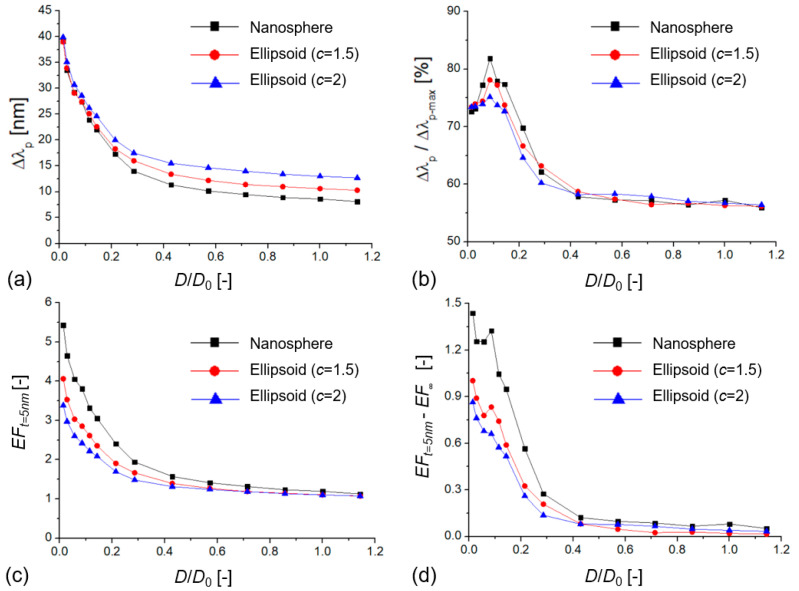
(**a**) Extinction peak shift ($\Delta \lambda_{p}$) of different dimer arrangements as a function of the dimensionless *D*/*D*_0_ value (*D*_0_ = 70 nm) with a dielectric layer of 5 nm (*n*_l_ = 1.5 in water medium, with *n* = 1.33). (**b**) Relative extinction peak shift as a function of the dimensionless *D*/*D*_0_ value, where $\Delta \lambda_{p-max}$ is calculated as the peak shift upon the RI of the medium changing from 1.33 to 1.5. (**c**) Enhancement factor (*EF*_t=5nm_) as a function of *D*/*D*_0_. (**d**) Difference between surface and bulk enhancement factors (*EF*_t=5nm_ − *EF*_∞_) as a function of *D*/*D*_0_.

## 2. Text Correction

There were two textual errors in the original publication.

On page 4, in one instance, *RIS*_dim_ was written instead of $\Delta \lambda_{p,dim}$.

A correction has been made to “3. Results and Discussion”, first paragraph:

“By depositing a dielectric layer of 5 nm thickness and *n*_l_ = 1.5, the experienced extinction peak shifts were $\Delta \lambda_{p,sp}$ = 6.1 nm and $\Delta \lambda_{p,dim}$ = 18.2 nm (Figure 2b), corresponding to an enhancement of 2.98 (*EF*_t=5nm_)”.

On page 6, in one instance, *t* = 7 nm was written instead of *t* = 5 nm. 

A correction has been made to “3. Results and Discussion”, seventh paragraph:

“The practical meaning of *EF*_t_ is, for example, that dimer nanospheres of 70 nm diameter and 10 nm gap provide a 3.2 times higher signal compared to uncoupled spheres of the same size with *t* = 5 nm layer thickness”.

The author apologizes for any inconvenience caused and states that the scientific conclusions are unaffected. The original publication has also been updated.

## References

[1] Bonyár A. (2021). Maximizing the Surface Sensitivity of LSPR Biosensors through Plasmon Coupling—Interparticle Gap Optimization for Dimers Using Computational Simulations. Biosensors.

